# Effect of intimacy and dyadic coping on psychological distress in pancreatic cancer patients and spousal caregivers

**DOI:** 10.3389/fpsyg.2023.1040460

**Published:** 2023-02-02

**Authors:** Jiarong Li, Linglong Liu, Mingxia Chen, Wang Su, Tianying Yao, Xiaoxuan Li

**Affiliations:** ^1^School of Nursing, Nanjing Medical University, Nanjing, China; ^2^School of Nursing, Faculty of Health, Queensland University of Technology, Brisbane, QLD, Australia

**Keywords:** pancreatic cancer, spouse, dyadic coping, actor-partner interdependence mediation model, anxiety, depression, intimacy, nursing

## Abstract

**Aims:**

The aim of this study was to investigate the effect of intimacy and dyadic coping on anxiety and depression in patients with pancreatic cancer and their spousal caregivers.

**Methods:**

This study conducted from October 2021 to June 2022, included 277 pancreatic cancer patients and their spousal caregivers. This research used actor-partner interdependence mediation model to explore the relationship of intimacy, dyadic coping, and psychological distress among pancreatic cancer patients and their spousal caregivers.

**Results:**

The results of this study showed that there were two actor effects: the satisfaction of intimate relationship between pancreatic cancer patients and their spouse caregivers had a positive predictive effect on their dyadic coping (*β* = 1.787, *p* < 0.001) and (*β* = 1.587, *p* < 0.001). The dyadic coping of pancreatic cancer patients and their spouse caregivers had a negative predictive effect on their own anxiety and depression (*β* = −0.253, *p* < 0.001) and (*β* = −0.293, *p* < 0.001). The results of this study showed that there was a partner effect: intimate relationship satisfaction of pancreatic cancer patients had a positive predictive effect on dyadic coping of their spousal caregivers (*β* = 0.574, *p* < 0.05).

**Conclusion:**

This study demonstrates the interdependence of pancreatic cancer patients and their spousal caregivers in coping with the disease. The healthy intimate relationship and effective dyadic coping styles are essential to alleviating disease pressure and lowering the psychological burden on cancer families.

## Introduction

Pancreatic cancer is a highly malignant gastrointestinal tumor. The 5-year survival rate is only 2–9% worldwide (Stathis and Moore, 2010), ranking the lowest among all malignant tumors (McGuigan et al., 2018). In China, its mortality rate ranks 7th among cancer-related deaths in the whole population (Sun et al., 2020). One study predicted that by 2040, the number of pancreatic cancer cases in China will increase by 190,532, which may be one of the largest increases in the number of countries (Hu et al., 2021). A study has shown that patients with pancreatic cancer had significantly lower QOL scores than healthy individuals (Carrato et al., 2015). Patients with pancreatic cancer, whose physical function is impaired, psychological status and social function are seriously affected, need caregivers to support them in disease and life care, especially their spouse as the main caregiver (Pistrang and Barker, 1995; Li et al., 2015).

A study from China showed that spousal caregivers accounted for 66.7% of informal caregivers and were the main caregivers of cancer patients (Li et al., 2018). Robinson conducted interviews with young cancer survivors in Canada to explore close relationships after a cancer diagnosis. It turns out that cancer can affect the intimacy of a couple, and this effect will continue throughout the course of cancer (Robinson et al., 2014). Intimacy are the product of interpersonal processes and are generated in the process of interaction (Harry, 2019). The process of intimacy begins when one person personally discloses feelings or information to another person, either verbally or non-verbally. The process continues when the listener is supportive or empathetic with the conveyor (Harry, 2019), which means that the intimacy are patterns of interdependence between subject and object. Particularly when cancer strikes an individual, cancer patients tend to convey negative emotions to their spousal caregivers who have a close relationship such as prognostic concerns and fear of recurrence. Thus, stressful events affect both partners, or one partner spreads their own stress into the close relationship and affects both partners. This stress is called dyadic stress (Bodenmann, 1995). In dyad, patients and spousal caregivers cope with the illness together, and the ways in which they support each other as they cope with cancer can affect their level of distress and quality of life. This common response and strategy in the face of stressful events is known as dyadic coping (Bodenmann, 1995, 2005). Bodenmann believed that dyadic coping mainly included two dimensions: positive coping and negative coping. The pressure communication, supportive coping, delegated dyadic coping, and common coping are main areas of positive dyadic coping, while negative dyadic coping includes hostile, ambivalent, and superficial (Bodenmann, 1995). A longitudinal study explored the dyadic coping and quality of life of patients with hematological malignancies and their partners through the APIM model, and found that their dyadic coping could affect their and their partners’ quality of life after 6 months (Ernst et al., 2017). In addition, dyadic coping has been shown to influence not only one’s own distress but also one’s partner’s distress (Banthia et al., 2003). This means that there are ways for couples to influence each other.

Kayser proposed the Relational-Cultural Coping Model (RCCM), which extends the earlier dyadic coping framework and views dyadic coping as a process (Kayser et al., 2007), and varies according to how couples assess disease-related stress, coordinate their coping efforts, and derive meaning from the illness experience (Kayser and Acquati, 2019). This model considered relational qualities of relational awareness, authenticity, and mutuality as important components of dyadic coping (Kayser et al., 2007). Relationship awareness refers to thinking about the impact of the illness on the partner and relationship in the context of the illness, authenticity refers to not hiding one’s true feelings from one’s partner, and mutuality refers to participating in an experience with one’s partner (Kayser et al., 2007). Couples with a good relationship will see illness as a common stressful event, as described by Kayser, using “we-disease”(Kayser et al., 2007). Kayser proposed this term for the first time in the study of qualitative interviews. In the process of data analysis, the subject of the topic was divided into two situations: “I” and “we.” “I-disease” means that couples see cancer as personal stress, only mention the impact of cancer on them, and usually deal with the disease in a disengaging and avoidant way.” We-disease” refers to couples perceiving disease stress as “we-disease” when assessing it, and using “We” as the subject to describe the disease during the interview(Kayser et al., 2007). Leuchtmann and Bodenmann extended the concept of “we-disease” in 2017. “We-disease” means a shared view of the illness and joint efforts of both partners (the patient as well as the other partner) to deal with the disease, and thus might lead to better outcomes for both partners(Leuchtmann and Bodenmann, 2017). So it could be argued that how close relationships are viewed also represents how individuals view the impact of illness on themselves and their partners. Kayser explored breast cancer patients and their partners through the APIM model and found that individual mutuality can affect the dyadic coping style of themselves and their partners (Kayser and Acquati, 2019). Couples with high levels of intimacy were more likely to communicate with each other about cancer and be more supportive of each other (Falconier et al., 2015), which could help them find an appropriate way to manage the emotional and physical demands of cancer.

The RCCM also pointed out that negative dyadic coping of cancer patients and spousal caregivers can bring some negative effects, such as anxiety and depression (Kayser and Acquati, 2019). Compared with other types of cancer, the psychological dimension of patients with pancreatic cancer is worse (Clark et al., 2010). Spousal caregivers are more likely than other family members to have anxiety and depression (von Heymann-Horan et al., 2018). Therefore, it is essential to explore the anxiety and depression of pancreatic cancer patients and spouse caregivers. A systematic review suggests that negative dyadic coping and protective buffering are associated with depressive symptoms (Chen et al., 2021). Facchin’s research has shown that better dyadic coping is associated with better mental health for women with endometriosis(Facchin et al., 2021). However, this study only analyzed the correlation of three variables, and did not deeply explore the path relationship. In addition, Cao explored the intimacy and depressive symptoms 2 years later in 203 Chinese couples and found that intimacy was associated with depressive symptoms in partners (Cao et al., 2017). In conclusion, there is an association between intimacy, dyadic coping, and anxiety and depression, which leads to speculation about whether there is a mediating relationship among the three.

All the above studies have proved that there is mutual influence between couples, and it is impossible to scientifically analyze paired data by using correlation study alone. Therefore, in this study, APIMeM model was used to study pancreatic cancer patients and partners. The actor-partner interdependence model (APIM) is an important method for analyzing dyad data, which can answer the question of whether an individual’s outcome variable is affected by his or her own predictor variables (Kenny and Ledermann, 2010). APIMeM is an extension of it to analyze the mediating effect, which can reveal the mechanism of the predictor variable affecting the outcome variable (Ledermann et al., 2011). In this study, it can be used to explore the path relationship between pancreatic cancer patients and spousal caregivers in the three variables.

Pancreatic cancer is currently an understudied group, especially as the incidence of pancreatic cancer increases and more and more couples suffer from the long-term stress of the disease. For this particular group, the level of intimacy between them is necessary to face complex and intractable complications and death threats together. If they are aware of the importance of the relationship and can use the relationship as an available resource, they will have a smoother subsequent coping with cancer. Therefore, to explore the relationship between close relationship, dyadic support coping, and anxiety and depression in pancreatic cancer patients and their spouse caregivers is of great significance for formulating dyadic interventions to effectively improve their quality of life.

## Materials and methods

### Design and participants

The study was a cross-sectional study conducted from October 2021 to June 2022. A total of 277 pairs of pancreatic cancer patients and their spousal caregivers in Pancreatic Center of Nanjing Jiangsu Hospital were selected as the research objects by convenience sampling. The general information questionnaire is shown in Table 1. It can be seen from Table 1 that most patients with pancreatic cancer are male and most of them are over 60 years old. Most of the patients are in stage 2, and the number of years of diagnosis is less than 2 years. About one-third of the patients and caregivers are retired.

**Table 1:** Descriptive statistics of pancreatic cancer patients and spousal caregivers.

Characteristics		Patients	Spousal caregivers
		*n*(%)	*n*(%)
Gender	Male	163(58.8)	114(41.2)
	Female	114(41.2)	163(58.8)
Age	30–39	4(1.4)	4(1.4)
	40–49	30(10.8)	27(9.7)
	50–59	83(30.0)	96(34.7)
	≥60	160(57.8)	150(54.2)
Level of education	Primary school or less	84(30.3)	74(26.7)
	Junior high school	123(44.4)	114(41.2)
	Senior high school	47(17.0)	67(24.2)
	University or above	23(8.3)	22(7.9)
Living condition	country	196(70.8)	
	City	81(29.2)	
Occupation	unemployed	24(8.7)	27(9.7)
	farmer	56(20.2)	59(21.3)
	entity	38(13.7)	40(14.4)
	employees of the enterprises	34(12.3)	30(10.8)
	Civil servants and public institutions	26(9.4)	29(10.5)
	retirement	99(35.7)	92(33.2)
Quantity of children	1	173(62.5)	
	2or more	104(37.5)	
Family monthly earning	<3000RMB	8(2.9)	
	3,000-5000RMB	102(36.8)	
	≥5000RMB	167(60.3)	
Religion	Yes	7(2.5)	5(1.8)
	No	270(97.5)	272(98.2)
Number of year of the sick	<1 year	96(34.7)	
	1–2 year	147(53.1)	
	2–3 year	22(7.9)	
	>3 year	12(4.3)	
Cancer staging	I	38(13.7)	
	II	144(52.0)	
	III	72(26.0)	
	IV	23(8.3)	

The inclusion criteria of patients were as follows: (1) Patients were pathologically diagnosed with pancreatic cancer; (2) Age ≥ 18; and (3) Informed consent and voluntary participation in this study.

The exclusion criteria of patients were as follows: (1) They had medical records showing a history of mental illness and dyslexia and (2) Combined with serious life-threatening diseases. Mental illness here mainly refers to schizophrenia, Alzheimer’s disease, etc., and this criterion is mainly set to screen out patients who cannot answer questions correctly. “Life-threatening diseases” refers to critically ill or terminally ill patients who may be unconscious and unable to respond to external stimuli. For such patients, the maintenance of vital signs is of utmost importance, and questionnaires would affect their treatment, so they could not be investigated. In addition, the medical history of the patients and their family members was inquired at the time of admission, and the presence or absence of dyslexia was recorded. Patients with dyslexia were unable to correctly read and respond to the questionnaire, which would have created a bias for this study, so these people were excluded.

The inclusion criteria of spousal caregivers were as follows: (1) husband and wife relationship with the patient; (2) Age ≥ 18; (3) Live with the patient, and the patient has designated the most time to care for them during the illness; and (4) Informed consent and voluntary participation in this study.

Exclusion criteria for spousal caregivers were as follows: having medical records showing a history of mental illness and dyslexia.

### Data collection

The investigators will communicate with the patients in advance, obtain their consent, and distribute questionnaires to the patients and their spouse caregivers who meet the inclusion criteria and agree to participate in the study. Investigators handed out questionnaires on site, and used unified instructions to explain to patients the significance of the survey, the time needed, and the matters needing attention. The questionnaire was completed by the patient and the spouse caregivers themselves. The researcher collected and checked the questionnaire on the spot, and checked with the patients and their spouse caregivers in time to ensure the completeness and accuracy of the questionnaire. A total of 284 pairs of questionnaires were distributed in this study, and 277 pairs of valid questionnaires were recovered.

### Measures

#### Demographic variables and clinical data

There were 10 items in the demographic variables and clinical data, including gender, age, place of residence, education level, number of religious children, family *per capita* monthly income, occupation, years of illness, and stage of cancer. The general information questionnaire of spouse caregivers consisted of 5 items, including age, sex, education level, religion, and occupation.

#### Chinese version of dyadic coping scale (DCI)

Bodenmann developed the Dyadic Coping Inventory, which included 6 dimensions of stress communication, supportive dyadic coping, negative dyadic coping, entrusted dyadic coping, common dyadic coping, and dyadic coping, with a total of 37 items (Bodenmann, 2008). The questionnaire was divided into five parts to investigate the research subjects: (1) How do you communicate your pressure with your partner, for example, I let my partner know that I appreciate his practical support, advice, and help. (2) What does your partner do when you are stressed? For example, my partner will tell me that he will stay with me. (3) What do you do when your partner is stressed? For example, I sympathize and understand my partner’s stress. (4) What to do when you and your partner are both stressed. For example, we face and solve problems together. (5) As a couple, how would you rate your coping style? For example, I am pleased with the support my partner provides and the way we cope with stress together. Except the dimension of negative dyadic coping is negative coping style, and the higher the score, the higher the level of negative dyadic coping, the other five dimensions reflect the positive coping style, that is, the higher the score, the higher the degree of positive coping. The Chinese version was translated into Chinese by Xu Feng in 2016, and the Cronbach’s alpha of each dimension was 0.51–0.80 in males and 0.52–0.80 in females (Xu et al., 2016). This scale can be used to evaluate the patient and spouse, respectively, which can accurately understand the mutual support of the patient and spouse. It is the most widely used assessment tool to evaluate dyadic coping behavior. In this study, the Cronbach’s alpha of DCI in pancreatic cancer patients and spousal caregivers was 0.728 and 0.830, respectively.

#### The quality of relationship index (QRI)

QRI was revised by Patrick et al. (2007). To investigate the individual’s perception and evaluation level of his/her current relationship with his/her lover, the questionnaire contains a total of 6 items, and the 7-level score is used, ranging from 1 completely disagree to fully agree. The scale includes things like we are in a good relationship, our relationship is stable, I am happy with my partner, and so on. The higher the score, the higher the individual’s satisfaction with the intimate relationship. The Cronbach’s alpha of the Chinese version was 0.916. In this study, the Cronbach’s alpha of QRI in pancreatic cancer patients and spouse caregivers was 0.765 and 0.836, respectively.

#### Hospital anxiety and depression scale (HADS)

This scale is a kind of self-rating scale, mainly used for rapid assessment of patients’ anxiety and depression, and is one of the tools for screening anxiety and depression (Johnston et al., 2000). The scale has 14 items, including 7 items for anxiety and 7 items for depression. The scale includes things like I am nervous (or miserable), I am still interested in things I used to be interested in, I am full of worries, I seem to be feeling low, and so on. Each item was scored at 4 levels from 0 to 3, and each subscale scored from 0 to 21. Generally, the cutoff value is divided into 7/8, >7 points had anxiety or depression symptoms, and 8 points were used as the cutoff value for screening in this study. The total score of 8 to 10 is mild anxiety/depression, 11 to 14 is moderate anxiety/depression, and 15 is severe anxiety/depression. The Cronbach’s alpha of the anxiety subscale of the scale was 0.84, and the Cronbach’s alpha of the depression subscale was 0.81. In this study, the Cronbach’s α of HADS in pancreatic cancer patients and spouse caregivers was 0.825 and 0.876, respectively.

### Ethical considerations

This study was approved by the Ethics Committee of Nanjing Medical University [ethics audit number (2022)763], and informed consent was obtained from all participants. Numerical coding was used for de-identification in this study.

### Data analysis

SPSS 21.0 and MPLUS 8.0 were used for statistical analysis. Mean standard deviation, frequency, and percentage (%) were used to describe general demographic data for pancreatic cancer patients and their spousal caregivers, including dyadic coping level intimacy and anxiety and depression scores. Pearson correlation analysis was used to analyze the pairwise correlation between dyadic coping, intimacy, and the total score of anxiety and depression. APIMeM was established using MPLUS to investigate the relationship between intimacy, dyadic coping, and anxiety and depression in pancreatic cancer patients and their spousal caregivers. Bootstrap method was used to test the mediating effect. Based on the two-tailed test, statistical significance was set at *p* < 0.05.

**Figure 1:**
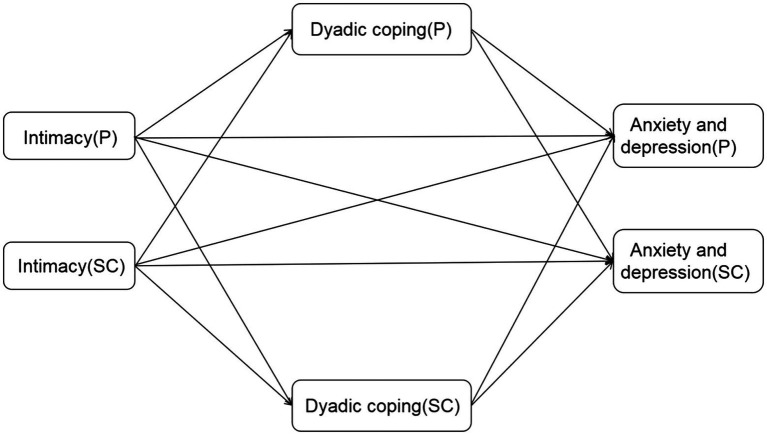
Actor-partner interdependence mediation model(APIMeM). P: patients; SC: spousal caregivers.

**Figure 2:**
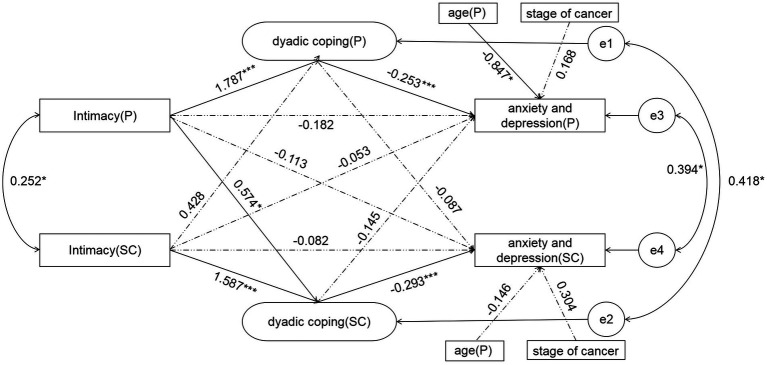
The final model of pancreatic cancer patients and spousal caregivers. *p < 0.05; **p < 0.01; ***p < 0.001.

APIMeM (Figure 1) is used to analyze the mediating effect of dyad data (Ledermann et al., 2011). Originally, APIM refers to the influence of an individual variable on his or her own outcome variable (actor effect) and the partner’s outcome variable (partner effect; Cook and Kenny, 2005). APIMeM extends this to relationships between three variables. In this study, the influence of the intimate relationship on anxiety and depression was explored, and dyadic coping was used as the mediating variable (Figure 2). In reviewing previous literature, researchers found that anxiety and depression in patients and spousal caregivers may be related to age and cancer stage. Therefore, age and stage of cancer were included as control variables in this study (Lee et al., 2013). The research steps are as follows: (1) first judge whether it is a distinguishable dyad; (2) If it is distinguishable dyad, a corrected saturation model is constructed to estimate the subject effect and partner effect, and then the total effect is calculated, direct effect and indirect effect; and (3) Confidence intervals of each effect value were obtained by bootstrap method. After correcting the saturation model to estimate the subject effect and the partner effect, the indirect effect and the total effect were obtained by adding the corresponding effect values.

## Results

### Common method bias test

Univariate Harman test was performed for variables such as dyadic coping and anxiety and depression. The results show that the maximum single-factor explained variance is 27.63%, which is less than 40% of the critical standard (Podsakoff et al., 2003). This indicates that there is no common method bias in this study.

### Mean scores for intimacy, dyadic coping, anxiety, and depression in dyads

The total score of intimacy of pancreatic cancer patients and their spouse caregivers was 38.08 ± 3.51 and 38.97 ± 3.43, respectively. The total scores of dyadic coping were 122.47 ± 12.98 and 121.97 ± 12.64, respectively. The total scores of anxiety and depression were 10.72 ± 12.98 and 10.62 ± 5.82, respectively.

According to Table 2, there was a significant difference in the score of dyadic coping (*p* < 0.05). But there was no significant difference in the scores of anxiety and depression (*p* = 0.407), and there was no significant difference in the score of intimacy between pancreatic cancer patients and spousal caregivers (*p* = 0.285).

**Table 2:** Patients and spousal caregivers’ scores on variables.

	Patients	Spousal caregivers	*t*	*p*
Intimacy	38.08 ± 3.51	38.97 ± 3.43	1.121	0.285
Dyadic coping	122.47 ± 12.98	121.97 ± 12.64	2.037	**0.026**
Anxiety and depression	10.72 ± 12.98	10.62 ± 5.82	0.831	0.407

Bold type indicates statistical significance (*p* < 0.05).

### Associations between intimacy, dyadic coping, and anxiety and depression

Table 2 shows that the scores of patients in dyadic coping and anxiety and depression are significantly positively correlated with the scores of their spousal caregivers (*p* < 0.001). The intimacy of spousal caregivers was positively correlated with dyadic coping scores (*p* < 0.001). Dyadic coping was negatively correlated with the scores of anxiety and depression (*p* < 0.001). The intimacy of patients was negatively correlated with the scores of anxiety and depression (*p* < 0.05). The intimacy of spousal caregivers was positively correlated with dyadic coping scores (*p* < 0.01). Dyadic coping of spousal caregivers was negatively correlated with anxiety and depression scores (*p* < 0.001). The intimacy of spousal caregivers was negatively correlated with anxiety and depression (*p* < 0.05). The association here is the association between the actor’s own two scores.

### Actor-partner interdependence mediation model

Taking the intimacy between pancreatic cancer patients and their spouse caregivers as the independent variable, dyadic coping as the mediating variable, and anxiety and depression as the dependent variable, APIMeM was constructed. Firstly, to determine whether pancreatic cancer patients and their spousal caregivers are distinguishable variables, the following constraints were created: aA1 = aA2; bA1 = bA2; cA1 = cA2; aP1 = aP2; bP1 = bP2; cP1 = cP2. Test whether the Chi-square value of the model changes significantly. In this study, *p* = 0.09, which is less than the suggested value of 0.2 given by Kenny and Ledermann (2010). Therefore, the two sides are regarded as distinguishable pairwise relationship. According to the model adaptation results, χ^2^/df = 1.67(<3), CFI = 0.997, TLI = 0.993, NFI = 0.963, AGFI = 0.974(>0.9), RMSEA = 0.031(<0.1). It indicates that the model fits well. Figure 2 illustrates the path model for pancreatic cancer patients and spousal caregivers. The dashed line indicates that the path is not significant.

Table 3 shows the values of direct, indirect, and total effects of patient and spousal caregivers. As shown in Figure 2 for the standard path coefficients, the patient’s intimacy could directly affect the dyadic coping of patients (*β* = 1.787, *p* < 0.001) and spousal caregivers (*β* = 0.574, *p < 0.05). The intimacy of spousal caregivers could only affect their own dyadic coping (*β* = 1.587, *p* < 0.001), but had no significant effect on the dyadic coping of patients, and there was no partner effect. The intimacy between patients and spousal caregivers can positively predict the dyadic coping of themselves and spousal caregivers. The dyadic coping of patients could directly affect their own dyadic coping (*β* = −0.253, *p* < 0.001), and the dyadic coping of spousal caregivers could affect their own dyadic coping (*β* = −0.293, *p* < 0.001), but there was no partner effect in both patients and spousal caregivers. The dyadic coping of patients and spousal caregivers can only negatively predict their own anxiety and depression. However, the intimacy between patients and spousal caregivers did not directly affect the anxiety and depression (Table 4).

**Table 3:** Correlations of the variables between pancreatic cancer patients and spousal caregivers.

	P1	P2	P3	SC4	SC5
P1:intimacy					
P2:dyadic coping	0.560^***^				
P3:anxiety and depression	−0.384^*^	−0.795^***^			
SC4:intimacy	0.179	0.441^**^	−0.339^**^		
SC5:dyadic coping	0.547^*^	0.977^***^	−0.794^***^	0.455^**^	
SC6:anxiety and depression	−0.419^*^	−0.797^***^	0.946^***^	−0.384^*^	−0.812^***^

*p < 0.05; **p < 0.01; ***p < 0.001.

**Table 4:** The direct, indirect, and total indirect effects for pancreatic cancer and spousal caregivers in the APIMeM.

Actor effects/partner effects	Estimate	S.E.	*p*
**Intimacy(P)-anxiety and depression(P)**			
Total effect	−0.501	0.132	**0.000**
Direct effect	−0.182	0.096	0.056
Total indirect effect	−0.683	0.100	**0.000**
**Intimacy(SC)-anxiety and depression(P)**			
Total effect	−0.244	0.132	0.063
Direct effect	−0.053	0.084	0.531
Total indirect effect	−0.192	0.091	**0.036**
**Intimacy(P)-anxiety and depression(SC)**			
Total effect	−0.509	0.124	**0.000**
Direct effect	−0.113	0.091	0.215
Total indirect effect	−0.622	0.092	**0.000**
**Intimacy(SC)-anxiety and depression(SC)**			
Total effect	−0.287	0.126	**0.023**
Direct effect	−0.082	0.087	0.347
Total indirect effect	−0.206	0.087	**0.018**

Bold type indicates statistical significance (*p* < 0.05).

### The mediating role of dyadic coping

Table 5 shows the mediating effect of dyadic coping. In the actor effects, dyadic coping played a mediating role between intimacy and anxiety and depression (*p* = 0.006). The dyadic coping of spousal caregivers played a mediating role between their own intimacy and anxiety and depression (*p* = 0.043). Among the object effects, the dyadic coping of spousal caregivers played a mediating role in the intimacy of patients and spousal caregivers’ anxiety and depression (*p* = 0.001).

**Table 5:** The mediating effect of dyadic coping and anxiety and depression in intimacy of patients with pancreatic cancer and spousal caregivers.

Actor effects/partner effects	Estimate	S.E.	*p*
Actor effects			
Intimacy(P)-dyadic coping(P)-anxiety and depression(P)	−0.452	0.164	**0.006**
Intimacy(P)-dyadic coping(SC)-anxiety and depression(P)	−0.231	0.136	0.091
Intimacy(SC)-dyadic coping(P)-anxiety and depression(SC)	−0.037	0.040	0.348
Intimacy(SC)-dyadic coping(SC)-anxiety and depression(SC)	−0.168	0.083	**0.043**
Partner effects			
Intimacy(SC)-dyadic coping(P)-anxiety and depression(P)	−0.108	0.069	0.119
Intimacy(SC)-dyadic coping(SC)-anxiety and depression(P)	−0.083	0.062	0.179
Intimacy(P)-dyadic coping(P)-anxiety and depression(SC)	−0.156	0.133	0.243
Intimacy(P)-dyadic coping(SC)-anxiety and depression(SC)	−0.465	0.134	**0.001**

Bold type indicates statistical significance (*p* < 0.05).

## Discussion

Due to cultural differences, couples in eastern countries tend to emphasize interdependence (Berg and Upchurch, 2007). When one party encounters difficulties, the other party should shoulder more responsibility. In dealing with the stress of the illness, cancer is not a personal event for the patient, and couples should see each other as a major source of support to get through it. This study is the first to explore the mediating effect of dyadic coping on intimacy and anxiety and depression in pancreatic cancer patients using APIMeM, which is helpful to deepen the understanding of the mechanism of partner intimacy on mental health status.

The study found that the psychological status of pancreatic cancer patients and their spouse caregivers were both mild anxiety and depression, and there was a significant correlation between them. This is the same result as a previous study (Janda et al., 2017). This suggests the need to study patients and spousal caregivers as a whole. While patients suffer from the pain caused by the disease, spousal caregivers also have the same distress, because they are faced with economic, occupational, emotional strain, and changes in family roles (Gremore et al., 2021). As many as 77 percent of patients and partners reported significant distress during treatment (Bodenmann and Randall, 2012). Due to the long time together and similar living habits, there is a certain consistency in physical and mental health between husband and wife, and they depend on each other and influence each other. Multiple studies have shown that couples are associated with a range of health conditions, for example, coronary heart disease, hypertension, and mental illness (Walker et al., 2017). Therefore, in the face of external pressures, patients or spouse caregivers cannot be considered separately from the dyad as a whole.

The relationship intimacy model believes that intimacy is an important factor affecting the psychological distress of cancer patients and spouses (Manne and Badr, 2008), and it is a resource that individuals can use in the face of stress. This study also confirmed the view of this model, the close relationship of pancreatic cancer patients and their spousal caregivers was negatively associated with anxiety and depression, which was also similar to the results of previous studies (Facchin et al., 2021). The findings support the idea that the greater the awareness of the relationship as a whole, the greater the commitment to happiness and love, and the greater the psychological adjustment. This study found that the close relationship between pancreatic cancer patients and their spouse caregivers was positively correlated with dyadic coping. Some researchers have analyzed the effect of cognition in intimacy on dyadic coping, and the results show that individuals who view stress as ours rather than mine are more likely to focus on shared coping in intimacy (Story and Bradbury, 2004). This has implications for reducing the psychological burden by increasing the intimacy of the couple. Badr conducted a trial of a couple-based communication intervention. It was found that patients and partners who started talking about the impact of lung cancer on their relationship early on were more likely to be better prepared together to deal with the challenges they might face, helping to reduce levels of psychological distress caused by the disease. In addition to couple-based communication interventions, there are psychoeducational interventions (Badr et al., 2008). For example, Chien taught couples diseases, emotional regulation, stress management, and identifying available social resources (Chien et al., 2020). Finally, the positive emotions of patients were improved, and the negative emotions of caregivers were improved.

In this study, two actor effect paths were found: intimacy(P) dyadic coping(P)-anxiety and depression(P); intimacy(SC)-dyadic coping(SC)-anxiety and depression(SC). Specifically, intimate relationship satisfaction of pancreatic cancer patients and their spouse caregivers positively predicted their own dyadic coping level, while dyadic coping level negatively predicted their own anxiety and depression. The higher the intimate relationship satisfaction of patients and their spouse caregivers, the more positive dyadic coping strategies they adopt, and the lower the level of anxiety and depression. This finding verified the role of intimacy in relationship culture coping model, suggesting that it is an important factor to promote dyadic coping (Kayser and Acquati, 2019). The analysis of the reason for this path may be that intimacy includes the emotional disclosure of the patient and the response of the other party. For the patient and the spouse, the emotional disclosure itself is conducive to the catharsis of emotion and the release of pressure (Harry, 2019). And the process of the spouse taking care of the patient is a process of maintaining high interaction. In this process, one partner communicates their feelings and information to the other, and the other partner responds by expressing sympathy or understanding, which encourages each partner to see the other not just as an “assistant,” but as a whole. Thus, couples with high interaction are more likely to adopt positive dyadic coping behaviors to support each other to overcome painful cancer experiences. The dyadic coping ability of the couple largely depends on the degree of previous interaction. Supportive behaviors such as comfort, advice, listening, and constructive criticism have positive effects on dyadic coping ability. When a couple is hit by the stress of illness, the way they have been together in the past can determine whether the couple sees cancer as our disease or my disease. This attitude toward illness affects the way couples cope with stress. Couples with high emotional interaction and trust in each other in daily life are more likely to view cancer as our disease. Adopting positive dyadic coping measures can help both partners manage disease stress and successfully improve their physical and mental health. However, if couples have less sympathy, support, and love in their close relationship, and cannot get interpersonal support in the face of disease pressure, it will lead to negative dyadic coping behavior and affect their own mental health. These findings suggest that dyadic coping style is a potential mechanism of relationship satisfaction affecting anxiety and depression, and improving the intimacy of couples and dyadic coping level is an effective measure to reduce anxiety and depression.

A number of studies have shown that DC is associated with reduced anxiety and depression symptoms. In Rottmann’s longitudinal study, patients who use negative coping styles seem to be doing more harm to themselves than to their partners (Rottmann et al., 2015). But Regan found that patients’ and wives’ use of supportive dyadic coping was not associated with their anxiety and depression; their partner’s use of this strategy was associated with their own anxiety and depression (Regan et al., 2014). This is different from the results of this study, in which patients’ binary coping only has subject effect but no object effect. These may indicate that: (1) patients are already suffering from the disease, especially pancreatic cancer patients, who suffer from the fear of surgical complications and high mortality. Having a negative coping style can do more harm than a spouse; (2) Even when providing supportive coping, patients were still focused on solving their own problems, suggesting that mental health problems may be more closely related to individual coping than dyadic coping.

The results of this study showed one partner effect: intimacy(P)—dyadic coping(SC)—anxiety and depression(SC). The intimacy satisfaction of pancreatic cancer patients positively predicted the dyadic coping level of caregivers, and then negatively predicted the anxiety and depression level of caregivers. The dyadic coping of spousal caregivers completely mediated the patient’s intimacy and the anxiety and depression of spousal caregivers. The intimate relationship of the partner only affects its own DC, while the intimate relationship of the patient affects its own DC and that of the partner. The reason may be that patients and their partners face different stressors. Illness stress is directly felt by the patients, and the partners are only indirectly affected (Kayser et al., 2007). Therefore, in order to reduce their own stress, patients adopt coping styles mostly based on their own feelings as the starting point. Therefore, patients’ dyadic coping was only influenced by themselves. Another possible explanation is that the main markers of intimacy are self-disclosure, sharing physical contact, sexual contact, appreciation of unconditional support, and so on (Yoo et al., 2014). Moreover, as the main source of support for cancer patients in the treatment process, spousal caregivers will react around the patients’ emotional feelings and expressions (Hodges et al., 2005). When the patients in a good intimate relationship exhibit the above behaviors, the spousal caregivers are more likely to think that the patients take positive coping measures, and less likely to take negative dyadic coping styles, which will eventually have a positive impact on their own mental health. This finding highlights the interaction between pancreatic cancer patients and spousal caregivers and suggests that a dyadic intervention based on the codependent nature of the couple can be used in future research to maximize both physical and mental health.

### Limitations

First of all, this study is a cross-sectional survey and cannot directly confirm the causal relationship between dyadic coping and anxiety and depression in pancreatic cancer patients and their spouses. Longitudinal studies can be carried out in the future to further explore the dynamic changes and interaction mechanisms among these variables. Secondly, the survey method of this study is self-filling scale, which may be biased to some extent. Third, this study is a single-center study, so it is impossible to explore whether there are different findings in different cultural and regional backgrounds. Fourth, this study did not divide dyadic coping into different dimensions, such as supportive, negative, delegated, and common.

## Conclusion

This study confirmed the dyadic relationship between pancreatic cancer patients and their spousal caregivers. In the process of coping with the impact of the disease, pancreatic cancer patients and spousal caregivers will make full use of marital resources to buffer the physical and psychological damage caused to them. It is emphasized that the importance of medical staff to take the patients and spousal caregivers as a whole when providing intervention for patients with pancreatic cancer, actively explore the interdependence between patients and spousal caregivers, and can use close relationship intervention to strengthen the awareness of the relationship between patients and spousal caregivers.

## Data availability statement

The raw data supporting the conclusions of this article will be made available by the authors, without undue reservation.

## Ethics statement

The studies involving human participants were reviewed and approved by the Ethics Committee of Nanjing Medical University [ethics audit number (2022)763]. The patients/participants provided their written informed consent to participate in this study. Written informed consent was obtained from the individual(s) for the publication of any potentially identifiable images or data included in this article.

## Author contributions

JL and WS: data collection. JL: statistical analysis and writing—original draft preparation. LL: statistical analysis and writing—review and editing. XL, TY, and MC: writing—review and editing. All authors contributed to the article and approved the submitted version.

## Conflict of interest

The authors declare that the research was conducted in the absence of any commercial or financial relationships that could be construed as a potential conflict of interest.

## Publisher’s note

All claims expressed in this article are solely those of the authors and do not necessarily represent those of their affiliated organizations, or those of the publisher, the editors and the reviewers. Any product that may be evaluated in this article, or claim that may be made by its manufacturer, is not guaranteed or endorsed by the publisher.
